# Comparative Study of *Pleurotus ostreatus* Mushroom Grown on Modified PAN Nanofiber Mats

**DOI:** 10.3390/nano9030475

**Published:** 2019-03-22

**Authors:** Lilia Sabantina, Franziska Kinzel, Thomas Hauser, Astrid Többer, Michaela Klöcker, Christoph Döpke, Robin Böttjer, Daria Wehlage, Anke Rattenholl, Andrea Ehrmann

**Affiliations:** Faculty of Engineering and Mathematics, Bielefeld University of Applied Sciences, 33619 Bielefeld, Germany; lilia.sabantina@fh-bielefeld.de (L.S.); franziska.kinzel@fh-bielefeld.de (F.K.); thomas.hauser@fh-bielefeld.de (T.H.); astrid.toebber@uni-bielefeld.de (A.T.); michaela.kloecker@fh-bielefeld.de (M.K.); christoph.doepke@fh-bielefeld.de (C.D.); robin.boettjer@fh-bielefeld.de (R.B.); daria.wehlage@fh-bielefeld.de (D.W.); anke.rattenholl@fh-bielefeld.de (A.R.)

**Keywords:** electrospinning, nanofiber mat, fungi, mycelium, *Pleurotus ostreatus*, composite, morphology

## Abstract

*Pleurotus ostreatus* is a well-known edible mushroom species which shows fast growth. The fungus can be used for medical, nutritional, filter, or packaging purposes. In this study, cultivation experiments were carried out with *Pleurotus ostreatus* growing on polyacrylonitrile (PAN) nanofiber mats in the presence of saccharose and Lutrol F68. The aim of this study was to find out whether modified PAN nanofiber mats are well suited for the growth of fungal mycelium, to increase growth rates and to affect mycelium fiber morphologies. Our results show that *Pleurotus ostreatus* mycelium grows on nanofiber mats in different morphologies, depending on the specific substrate, and can be used to produce a composite from fungal mycelium and nanofiber mats for biomedical and biotechnological applications.

## 1. Introduction

In recent years, electrospinning technology has become more and more popular for the production of nanofiber mats. The possibility to produce textile fabrics out of nanofibers has attracted interest in various areas of science, such as tissue engineering and regenerative medicine, with a rapid increase in recent years [1,2,3,4]. This technology offers several advantages which enable the growth of eukaryotic cells on nanofibrous textiles. Surface morphology is an important factor for the adhesion and spreading of cells, offering numerous adhesion points for cells to grow [5,6,7].

Besides applications in tissue engineering, the large surface-to-volume ratio makes nanofiber mats also suitable as filters. In addition, their high porosity and permeability combined with small pore size allows for reliable filtering of the finest particles [8,9,10].

Other possible fields for application of nanofiber mats as filters include optical and chemical sensors, nanocatalysis, energy storage, defense, aerospace, transportation, protective clothing, air, and water filters for medical and biotechnological applications, as well as dye filters for the textile finishing industry [11,12,13,14,15,16]. Despite their advantages, there is also one big problem which limits the use of nanofiber mats as filters: the mechanical weakness of single nanofiber mat layers [9,17,18]. While this problem is usually addressed by forming composites with macroscopic textile fabrics to create mechanically stable filters, connecting multiple nanofiber mats [19], or introducing nanofiber mats into sponge-like structures [20], another possibility could be combining nanofiber mats with biological stabilizing structures.

Similar to the above-described cell growth on nanofiber mats for tissue engineering and other biotechnological applications, other biological structures with higher intrinsic (i.e., not achieved by an additional coating, embedding of nanofibers, etc.) stability could be suitable for increasing the mechanical properties of nanofibrous filters in comparison with the pure nanofiber mat, such as self-assembling plant cells, which are able to dry and to form a mechanically stable state. Another biological material well-known for its good mechanical properties is the mycelium of different fungi. Usually, reports about fungicide properties of diverse nanofiber mats can be found in the scientific literature dealing with nanofiber mats and mentioning fungi [21,22,23,24,25]. Only very few studies investigate interactions between fungi and nanofiber mats in which the fungi grow on polymeric material. Ohkawa et al. found that different filamentous fungi were able to support biodegradation of electrospun poly(epsilon-caprolactone) nanofiber mats, allowing the biodegradability of this material to be tailored toward different environmental applications [26]. Even more interesting, Spasova et al. managed to electrospin *Trichoderma viride* spores in a chitosan solution and showed that these incorporated spores were still viable after this process, allowing them to grow and reproduce normally [27]. In a recent publication, the effect of electrospun nanofibers from cellulose acetate, cellulose, polyacrylonitrile (PAN), PAN with graphene, PAN–polymethyl methacrylate, and PAN–polyethylene glycol on the growth behavior of yeast cells was described [28].

Nevertheless, no other reports on fungal growth on nanofiber mats can be found in the literature. While there are several reports on the ideal growth conditions of, e.g., the edible oyster mushroom *Pleurotus ostreatus*, the substrates used in these growth tests are typically sawdust [29,30], although one study has shown the strong influence of substrate on *Pleurotus ostreatus* growth [31].

The chitin in the cell walls of *Pleurotus ostreatus* mycelium [32] makes it a promising candidate for the mechanical stabilization of nanofiber mats for filters and other applications, including tissue engineering and in batteries. Typical electrospun filters are produced from water-stable polymers, such as polyacrylonitrile (PAN) [9,33,34,35] or other different polymers. PAN, however, does not offer any nutrients for fungal growth. This is why, similar to [27], sugar was added as a possible nutrient. In addition, tests were performed by adding the poloxamer Lutrol F68 to PAN, which has been shown to be useful in typical biomedical applications, such as drug delivery [36], skin tissue engineering [37], or wound healing [38], and was also shown to strongly vary the nanofiber morphology [39]. Additionally, it may be regarded as sacrificial material which is washed out of the nanofiber mat when in contact with water and, in this way, enlarging the porosity of the nanofiber mat. Finally, stabilized PAN nanofiber mats were investigated as substrates, offering a possibility to functionalize the composite filters by carbonization. In addition, stabilized nanofibers are known to change their morphology by connecting fibers at crossing points [40], which may have an influence on the morphology of the mycelium.

This article provides first impressions of the growth of *Pleurotus ostreatus* mycelium on nanofiber mats, examining the possibility of using electrospun substrates and to create bio-based composites in this way. The study focuses on investigations of the general effect of nanofiber mats as substrates, especially with respect to morphology, while tests of the mechanical properties of the composites in dry and wet conditions will be carried out in a future study.

While this first investigation concentrates on the influence of the substrate on the mycelium morphology, serving as basic research for tests of growth rates and mechanical properties, the final technical applications can be expected to be filter materials with increased mechanical properties due to naturally built composites from nanofiber mats and the mechanically more-stable mycelium fibers. However, to reach this goal, basic tests of mycelium growth morphology—including investigating whether the mycelium grows through nanofiber mats and can thus be expected to automatically form a composite—are necessary, and are reported in this paper.

## 2. Materials and Methods

Nanofiber mats were produced on a polypropylene nonwoven as substrate using the needleless electrospinning machine Nanospider Lab (Elmarco Ltd., Liberec, Czech Republic). The following spinning parameters were used for production: high voltage, 70 to 80 kV; nozzle diameter, 0.8 mm; carriage speed, 100 mm/s; bottom electrode/substrate distance, 240 mm; ground electrode/substrate distance, 50 mm; temperature in the chamber, 22 °C; relative humidity in the chamber, 32%. Spinning was carried out for 30 min. These spinning parameters were found to be ideal in former electrospinning experiments with PAN [41,42]. High voltages in the range of 70–80 kV would be unusual for needle-based electrospinning, where voltages typically in the range of 20 kV are used. For the wire-based technique applied here, PAN can be spun at voltages higher than approximately 50 kV, but even higher voltages result in thicker nanofiber mats and are thus advantageous in most cases [19,33,40,41,42].

The spinning solution for the production of nanofibers contained polyacrylonitrile (PAN) (Woolworth, Unna, Germany) dissolved in DMSO (dimethyl sulfoxide, min 99.9%, purchased from S3 Chemicals, Bad Oeynhausen, Germany). DMSO was chosen as the solvent because it is non-toxic [43,44]. The following protocols were used to create three different nanofiber mats using PAN solid contents that were found to be ideal in former experiments for pure PAN, or in combination with poloxamer [41,42,45]:

(A) In a solution of 16% PAN in DMSO (18 g), 20 g saccharose (food grade, Pfeiffer & Langen GmbH & Co. KG, Cologne, Germany) were dissolved prior to electrospinning.
(B) 11.6% PAN + 13% poloxamer “Lutrol F 68”, 7680-9510 Da, 2 × 40% hydrophilic parts, sol–gel transition temperature approx. 45 °C [46] (BASF, Ludwigshafen am Rhein, Germany).
(C) 16% PAN in DMSO, stabilized at 280 °C for 1 h after electrospinning, heating rate 1 K/min, in a B150 muffle furnace (Nabertherm, Lilienthal, Germany).

All solutions were prepared by stirring the polymer solution for 2 h on a magnetic stirrer at room temperature.

Malt extract agar was used as nutrient medium for mushroom mycelium, which was produced from 1 L deionized water, 24 g agar (Agar-agar Kobe I, Roth, Karlsruhe, Germany), 20 g barley malt extract (Lindenmeyer GmbH & Co. KG, Weinsberg, Germany), and 1 g peptone (peptone water 77185, Sigma-Aldrich GmbH, Steinheim, Germany). After production, the fluid solution was poured into 8 cm diameter glass petri dishes (MSG, Wuppertal, Germany) and autoclaved for 20 min at 121 °C in an autoclave Systec-VX75 (Systec, Linden, Germany).

Saccharose serves as an additional nutrient only in recipe A, while in recipes B and C, malt agar extract is used to supply nutrients to the mycelium [47].

In addition, pure malt agar—without nanofiber mat—was used as a reference for mycelium growth.

**Figure 1:**
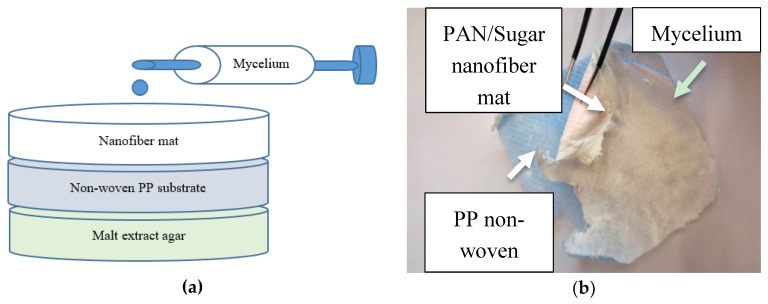
Growth of oyster mushroom mycelium on nanofiber mats: (**a**) schematic experimental setup; (**b**) example of inoculated specimen with fungal mycelium grown on a PAN/sugar nanofiber mat after its removal from the malt extract agar layer.

The autoclaved petri dishes with malt agar mixture were then inoculated with a sterile liquid mycelium syringe “oyster mushroom culture XXL, BIO” (purchased from Mushrooms & Equipment Shop, Münster, Germany), each with a 1 mL liquid mycelium culture. The schematic of the experimental setup of samples is shown in Figure 1.

After all petri dishes were provided with mycelium, they were sealed with Parafilm (Pechiney Plastic Packaging, Chicago, IL, USA) and stored at room temperature or at different temperatures, as described below, in the dark. Every two days, the hyphae were checked to see whether they had started growing and spreading radially. *Pleurotus ostreatus* growth was terminated by inactivation in the oven at 60 °C for 1 h.

To examine the general possibility of carbonizing nanofiber mats and mycelium together to create carbon composites, some of the PAN/mycelium composites were chemically stabilized by heating to 280 °C for 1 h, at a heating rate of 1 K/min, and afterwards, carbonized at 500 °C for 1 h in an SR (A) tube furnace (heating rate, 10 K/min; nitrogen flow, 150 mL/min; Carbolite Gero, Neuhausen, Germany). It must be mentioned that no optimization of the stabilization and carbonization temperatures and heating rates was performed especially for the mycelium, but optimal values of PAN and PAN/gelatin nanofiber mats were used [40].

For the optical examination of samples, a confocal laser scanning microscope (CLSM) VK-9000 (Keyence, Neu-Isenburg, Germany) with a nominal magnification of 2000× was used. Scanning electron microscopy (SEM) Zeiss 1450VPSE (Oberkochen, Germany) was applied for more detailed examinations of the fiber surfaces and morphologies. Nanofiber diameters were investigated using the software ImageJ 1.51j8 (from National Institutes of Health, Bethesda, MD, USA) on 50 fibers per sample.

## 3. Results

Nanofiber mats prepared from PAN/saccharose, PAN/poloxamer, and pure PAN after stabilization showed fiber diameter distributions of 345 ± 79, 530 ± 70, and 196 ± 80 nm, respectively. PAN/poloxamer nanofiber mat morphologies were not influenced by watering, thus, the original idea of using water-soluble poloxamer as a possible sacrificial material could not be verified. Instead, this blend formed solid fibers.

**Figure 2:**
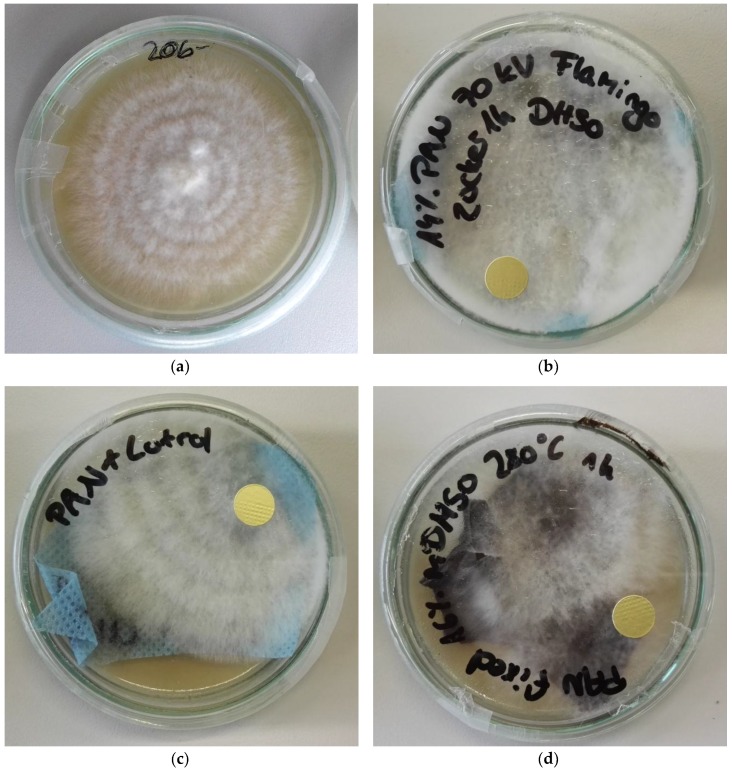
Oyster mushroom mycelium grown on different substrates, with images taken after 10 days: (**a**) malt agar extract; (**b**) PAN/saccharose nanofiber mat on malt agar extract; (**c**) PAN/poloxamer nanofiber mat on malt agar extract; (**d**) stabilized PAN nanofiber mat on malt agar extract.

As a basis for the evaluation of mycelium growth on different nanofiber mats, first tests were performed growing *Pleurotus ostreatus* on malt agar in petri dishes. The experiments showed the highest mycelium growth for environmental temperatures of approximately 25 °C, typically resulting in the petri dishes being completely covered with a fine layer of mycelium after 10 days (Figure 2a). It should be mentioned that at a growth temperature of 25 °C, all 5 samples were completely covered with mycelium, while at a temperature of 20 °C, 1 of the 5 samples was not yet fully covered, and at a higher growth temperature of 30 °C, mycelium growth was visible on only 3 of the 5 samples. Due to these observations, the next tests were performed at room temperature (~22–23 °C). As can be seen in Figure 2a, mycelium growth on agar is always radially oriented. This was also found by other researchers [48], while mycelium growth on wood, for example, is more longitudinally oriented [49].

Mycelium growth was also investigated on different nanofiber mats, as depicted in Figure 2b–d. Firstly, it can be seen that on the pure malt agar, the mycelium grows more densely on the nanofibers and only reluctantly across the fiber mat rims. Especially for PAN/sugar (Figure 2b) and PAN/poloxamer (Figure 2c), it is clearly visible that the mycelium layer is denser than in the case of pure agar (Figure 2a). The thickness of the mycelium varies strongly between approx. 1 mm and the maximum height which is limited by the closed petri dish. The stabilized nanofiber mat was partly broken, which seems to impede mycelium growth (Figure 2d). This corresponds with the above-described finding that the mycelium grows denser on nanofiber mats. Since no former investigations of mycelium growth on nanofiber mats can be found in the scientific literature, this behavior can only be assumed to be correlated with the surface structure, since it is known that the natural substrate for this mycelium is wood, whose structure may be mimicked better by the nanofiber mats than by the flat, even malt agar surface. These first images suggest that mycelium growth is not only possible on different nanofiber mats, but the material yield may even be increased by electrospun substrates, compared to using pure agar as substrate. This finding is important for applications requiring large amounts of mycelium, and will be investigated in more detail in a larger future study. It should be mentioned that on this macroscopic scale, growth on PAN/poloxamer and stabilized PAN seems to be, again, oriented linearly in a radial direction, while no such preferred orientation is visible for PAN/saccharose (Figure 2b).

**Figure 3:**
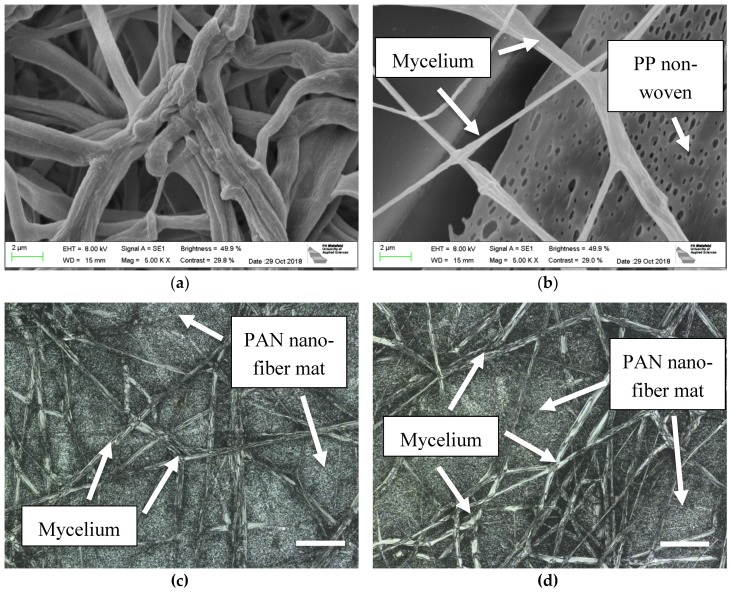
SEM images of oyster mushroom mycelium (**a**) grown on agar; (**b**) grown between a PAN nanofiber mat and the nonwoven PP used as a typical support for electrospinning; scale bars indicate 2 µm; CLSM images of (**c**) mycelium on PAN nanofiber mat; (**d**) mycelium under PAN nanofiber mat, being grown through it. Scale bars indicate 20 µm.

Here, however, another aspect is in the focus of the investigations. Figure 3a shows a typical mycelium structure, grown on the usual malt agar substrate, while Figure 3b depicts a few mycelium fibers left after pulling the nanofiber mat from the polypropylene (PP) support on which electrospinning usually occurs. The PP substrate was not detached before the growth test from the nanofiber mat since we tried to avoid breaking of the latter, while detaching afterwards was performed to investigate whether the mycelium grew through the nanofiber mat, which could be verified. The mycelium morphology seems to differ between the irregular, knotty structures grown on agar and the straight, even fibers grown on and under the nanofiber mat. It should be mentioned that the irregular, knotty structure of mycelium grown on agar, in the nanoscale, does not correspond to the clearly linear radial growth in the macroscale, as seen in Figure 2a.

Figure 3c,d depict CLSM images of the mycelium grown on the PAN nanofiber mat (Figure 3c) as well as through it, as visible from below (Figure 3d). In both cases, the thicker mycelium fibers can clearly be distinguished from the thinner nanofibers in the mat. The mycelium grows relatively straight and, as clearly seen in both images, not only through the mat, but even inside it, i.e., parallel to the nanofiber mat surface. This underlines the possibility of using mycelium to increase the mechanical properties of nanofiber composites, as compared to pure nanofiber mats.

**Figure 4:**
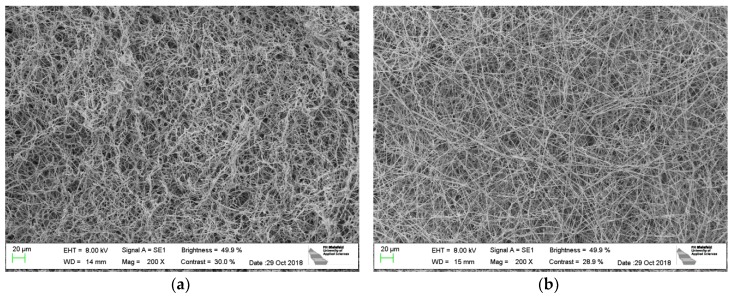
SEM images of oyster mushroom mycelium on different nanofiber mats: (**a**) PAN/poloxamer; (**b**) stabilized PAN without further additives. Only the mycelium is visible, completely covering the nanofiber mats below. The nominal magnification is 200×. Scale bars indicate 20 µm.

This finding was investigated in more detail by comparing mycelium growth on different nanofiber mats. Figure 4 shows, exemplarily, mycelium grown on PAN/poloxamer (Figure 4a) and on stabilized PAN (Figure 4b), respectively. Here, again, the morphologies clearly differ between the straight, even mycelium fibers grown on the stabilized PAN and the more chaotic, irregularly bent fibers grown on PAN/poloxamer. This finding is unexpected, since the stabilized PAN shows more conglutinations at fiber crossing points, as mentioned above [35,50], which does not intuitively indicate the formation of straighter mycelium fibers on this substrate. Apparently, the mycelium morphology does not only differ from agar to nanofiber mats as substrates, but even between different nanofiber mats. It must be mentioned that this finding was also not expected based on the macroscopic images where mycelium on PAN/poloxamer showed a clearly linear, radially oriented growth (Figure 2c). Apparently, it is necessary to distinguish between nano- and macrostructure for the description of mycelium growth.

**Figure 5:**
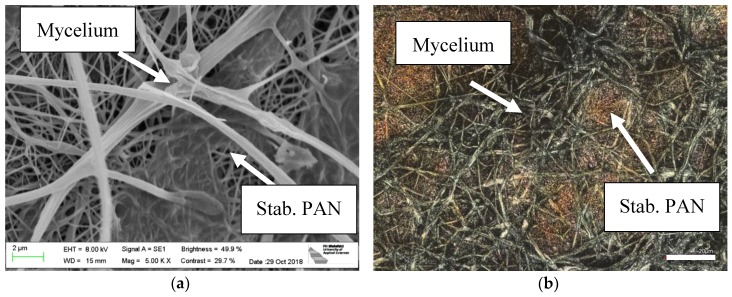
Oyster mushroom mycelium on stabilized PAN without further additives: (**a**) SEM image, scale bar indicates 2 µm; (**b**) CLSM image, scale bar indicates 20 µm. Thicker grey fibers are created by the mycelium, while thinner brown fibers stem from the stabilized nanofiber mat.

To investigate the unexpected finding that mycelium fibers grow straighter on stabilized PAN, Figure 5a depicts the mycelium fibers grown on stabilized PAN again at a higher magnification, while Figure 5b clearly shows the differences between the brown (=stabilized) PAN and the grey mycelium. Both images exhibit not only that the mycelium fibers grow through the openings between the nanofibers and can thus form a composite but, again, indicate that the mycelium fibers have a very straight morphology, making them more mechanically stable than the curling fibers growing on agar or on PAN/poloxamer, since the straight fibers cannot be lengthened by a force along their axes.

**Figure 6:**
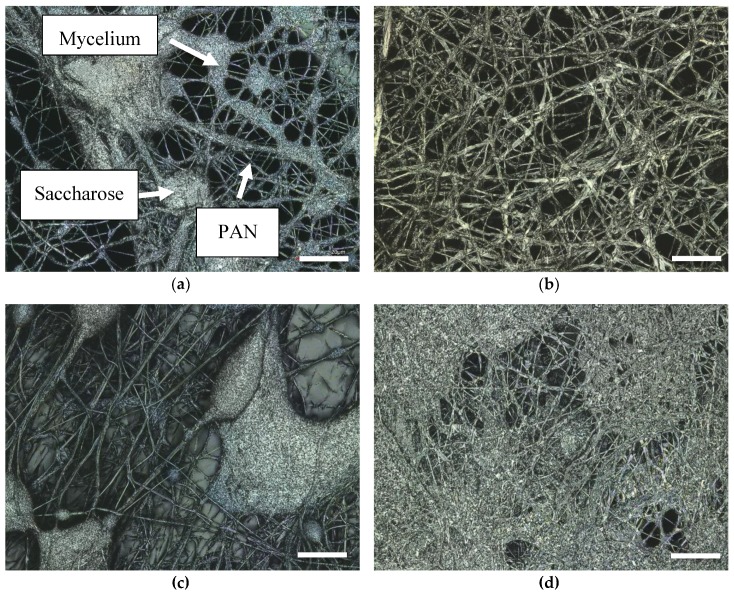
CLSM images: (**a**) oyster mushroom mycelium (thick fibers) on PAN/saccharose nanofiber mat (thin PAN fibers and saccharose agglomerations); (**b**) oyster mushroom mycelium on PAN/poloxamer nanofiber mat (the latter not visible here); (**c**) and (**d**) PAN/saccharose nanofiber mats after watering. Scale bars indicate 20 µm.

Figure 6a shows mycelium grown on a PAN/saccharose sample, which clearly differs from the other samples. Here, small thickenings and conglomerations can be observed, which are not visible on samples with PAN/poloxamer (Figure 6b) and stabilized PAN (Figure 5b) nanofiber mats. To investigate whether these conglomerations consist of sugar or of mycelium, Figure 6c,d show different positions on PAN/saccharose nanofiber mats after watering. Apparently, round or ellipsoidal conglomerations were formed after watering (Figure 6c), but the sugar partly also washed off (Figure 6d), so that it can be concluded that the conglomerations in Figure 6a most probably consist of saccharose.

The fiber structure of a PAN/saccharose mat seems to be more irregular than that of the other two nanofiber mats. Apparently, the sugar influences mycelium formation in a different way than the stabilized PAN nanofiber mats or PAN/poloxamer mats do. Nevertheless, the strong percolation of the mycelium through the nanofiber mats is, again, visible, underlining the possibility of forming composites in this way.

**Figure 7:**
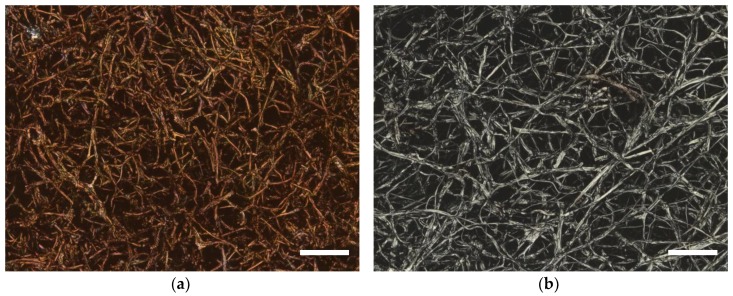
CLSM images of oyster mushroom mycelium on PAN nanofiber mats (the latter not visible) (**a**) after stabilization of the whole composite for 1 h at 280 °C; (**b**) after carbonization for 1 h at 500 °C of the whole composite. Scale bars indicate 20 µm.

Finally, it was tested whether it would also be also possible to stabilize and carbonize a nanofiber mat together with mycelium. To avoid confusion with carbonized sugar or poloxamer, the fungus was grown on pure PAN nanofiber mats (16% PAN dissolved in DMSO) prior to stabilization. CLSM images of the stabilized and carbonized mycelium are depicted in Figure 7. Unexpectedly, the mycelium shows less straight structures than in the previous tests on stabilized PAN (Figure 5). Apparently, the stabilization process changes the mycelium morphology, similar to the well-known change of the nanofiber structure during stabilization [40]. This has to be examined, in detail, in a future study. The mycelium fiber diameters are again, as in the experiments before, in the range of approx. 0.5–3 µm, with most fibers having diameters in the range of 1–2 µm.

The experiment shows, however, that stabilization and carbonization of oyster mushroom mycelium grown on PAN nanofiber mat is possible. In this way, pure carbon composites consisting of thicker mycelium fibers and thinner nanofibers can be realized, paving the way to carbon composites from fibrous structures of different diameters.

## 4. Discussion

Our experiments have given new insights into possibilities to grow the oyster mushroom *Pleurotus ostreatus* on nanofiber mats. Since this oyster mushroom is of high industrial interest due to its edible fruiting body and ability to accumulate selenium, an important essential trace element [51], several studies are reported in the scientific literature that deal with the influence of the substrate and other growth conditions on the chemical composition of the fruiting body and on mycelium morphology. Just recently, a study was published on *P. ostreatus* and another mushroom growing on different substrates and forming composites with them [52]. The authors showed that mycelium grown on sawdust as a substrate had a higher density than mycelium grown on straw or cotton fibers, while the mechanical properties were found to be dependent only on the following fabrication process to form a composite. This demonstrates, similar to our study, that the substrate influences the density of the mycelium grown on it, while in this study, no investigations of the original mycelium structure before pressing were performed. A possible dependence of the substrate on mycelium morphology was not evaluated.

Likewise, most other studies examined only mycelium growth without taking into account the morphology of the mycelium. In a detailed study, Dzulkefli and Zainol investigated the mycelium extension rate for mushroom cultivation on empty palm fruit bunches or sugarcane bagasse as substrates and its dependence on different mass ratios of spawn to substrate, substrate size, growth temperature, and a possible steam pretreatment [53].

Only a few other studies have mentioned the morphology of the mycelium. Haneef et al. cultivated *P. ostreatus* on cellulose and cellulose/potato dextrose, respectively. The authors found different amounts of polysaccharides, proteins, chitin, etc. in the mycelium, depending on the substrate. They concluded that the mycelium became stiffer on a harder-to-digest substrate, such as cellulose, as opposed to the small sugar molecules of potato dextrose [54]. This result fits with the findings of our study, that the mycelium grown on saccharose-coated nanofiber mats shows a different structure than the mycelium grown on pure or stabilized PAN (cf. Figure 2 and Figure 6).

Investigations of the mycelium morphology of other mushroom species also revealed differences between vegetative and generative development stages [55], a point which has to be taken into account in future long-term studies of *P. ostreatus* mycelium growth. Mykchaylova et al. found a dependence of the mycelium morphology of *Fomitopsis officinalis* on the nutrient medium [56]. Bellou et al. attributed differences in the morphology of *Yarrowia lipolytica* to the dissolved oxygen concentration, defining whether mycelium or cells with yeast-like morphology are developed [57].

The latter mushrooms, however, have properties very different from the here-examined *P. ostreatus*, and should thus be kept in mind for future examinations, but cannot be directly compared with the results of our study.

Stabilization and carbonization of the *P. ostreatus* mycelium, which was shown here for the first time, clearly influenced mycelium morphology, suggesting that further investigations on the process parameters are warranted.

## 5. Conclusions

Here, we report on first successful experiments growing oyster mushroom (*Pleurotus ostreatus*) mycelium on PAN nanofiber mats, partly with additional ingredients. The underlying nanofiber mats enable tailoring of mycelium morphology, which in turn allows for modifying the mechanical properties. On the other hand, the complete PAN/mycelium composites can be stabilized and carbonized, thus allowing for the creation of carbon composites with different fiber dimensions.

Following this first proof-of-principle, further experiments will examine the influence of nanofiber morphology and chemical composition on the mycelium growth and morphology, its mechanical properties, as well as the overall carbon yield.
